# Some Practical Thoughts on the Development of the Human Race and Obstetric Nursing

**Published:** 1897-04

**Authors:** R. R. Kime

**Affiliations:** Atlanta, Ga., Ex-President Atlanta Obstetrical Society; Ex-President Atlanta Society Medicine; Ex-Vice-President Tri-State Medical Society of Georgia, Alabama, and Tennessee


### SOME PRACTICAL THOUGHTS ON THE DEVELOPMENT OF THE HUMAN RACE AND OBSTETRIC NURSING.

BY R. R. KIME, M.D., ATLANTA, GA.,

Ex-President Atlanta Obstetrical Society; Ex-President Atlanta Society Medicine; Ex-Vice-President Tri-State Medical Society of Georgia, Alabama, and Tennessee.

(*Continued from March Number.*)

Very few mothers recognize the necessity for *strict surgical cleanliness* at these times and at labor, or fully understand the dangers of infection. In city life the bad air, imperfect sewerage, poorly kept water-closets, crowded, illy ventilated apartments, close association with persons who have been with infectious diseases, with the increased prevalence of venereal diseases, all conspire to render childbirth more hazardous than in the country.

City women, reared in luxury and idleness, without physical exercise, taxed mentally, with nervous system kept at high tension by manner of life, education (so-called), and the demands of fashionable society, are poorly developed physically, and not prepared for matrimony, much less maternity.

With imperfectly developed osseous, muscular and nervous system, they are favorable subjects for the invasion by disease, especially infection at childbirth.

The generative organs also being imperfectly developed, they are more liable to accidental complications and injury during labor and retarded involution (return to normal) after labor.

Women are not alone responsible for all the evil in the world, nor for diseases occurring as a result of childbirth. The husband may cause serious trouble by his indiscretion, contracting disease by immoral conduct, and infect his wife before or after childbirth, thus causing disease and suffering, not only of the one who should be dearest to him, but of his offspring also.

All venereal diseases of husband or wife are of serious import in connection with pregnancy or child-birth, and should be thoroughly eradicated before marriage, and especially before maternity is thought of. Many cases of invalid wives and diseased children, when properly interpreted, are but constant reminders of past sins.

While these factors add to the sum total of danger, the majority of diseases and accidents during the lying-in period, especially infections, are preventable by proper care and diligence upon the part of the mother, nurse, and physician.

Frequently trouble in these cases, especially infection, is due to neglect, ignorance, or inefficiency on the part of the mother or nurse, and the physician is held responsible when others are to blame.

In the selection of a *nurse* and *attendants* the mother cannot be too cautious, as her life frequently depends on the selection.

Many a woman has had serious trouble and some die as a result of ignorance, negligence, and want of cleanliness upon the part of the nurse, or as a result of her own preparation of clothing, bedding, dressings, room, and utensils to be used during confinement. An *old feather-bed* handed down as an heirloom is but a death-trap. A *syringe* so good that all the neighbors have used it is an abomination and as dangerous as dynamite. None but a new syringe, *or one that has not been about any infectious disease,* should ever be used, and not even the latter until it is thorougly washed, scalded, disinfected, and the nozzle boiled. The *bed-pan* is a deadly enemy, and should never be used in any case until thoroughly washed, scrubbed, boiled and disinfected, especially when used by the neighbors. *Sponges* for cleansing off discharges are hot-beds for infection, and should never be used, nor cloths be used a second time.

No woman should be confined in a bed or bedroom where there has recently been cases of septic infection, erysipelas, gangrene, smallpox, diphtheria, scarlet fever, measles, or any severe form of suppuration. The room and contents, bed and bedding, used about such cases should be thoroughly disinfected before being used.

By many women old quilts, blankets, cloths, etc., are selected for the occasion because they save expense and can be thrown away after use. Such things are extremely dangerous, for in them lurk the germs of disease, which may cause serious infection and death when they are used without disinfection or at least washing and boiling them. All sheets, blankets, old quilts, cloths, patient's clothing—in fact, everything that comes in contact with the body of the patient, especially such articles as have been used or soiled, should be washed and boiled in a closed vessel so as to retain part of the steam, thus destroying all infectious germs.

*Old cloths used to catch the discharges (lochia), without this boiling, are dangerous, have infected many a woman and killed not a few.*

*Diseases that are considered infectious at childbirth:* Septicemia (so-called "puerperal fever," or "childbed fever"), smallpox, diphtheria, scarlatina, measles, *gangrene, erysipelas*, surgical fever, discharge from carbuncle, abscess, or any purulent discharge from suppurating sores or diseased bones.

### NURSE.

A dirty nurse carries germs, which develops disease. A clean nurse destroys germs, which prevents disease.

A careless nurse neglects details, and carries the insignia of mourning under her finger-nails.

A careful nurse attends to details with clean hands and finger-nails, bringing health and happiness by aseptic methods.

The nurse is second only to the physician in responsibility, and may save the life or cause the death of the patient. She should be clean, intelligent, and understand the necessity of cleanliness in these cases; know how, or *willing to be instructed how, to keep patient surgically clean.*

She should not wear any clothing worn while nursing a previous infectious case (see list), unless they have been properly disinfected. No one should be allowed to enter the room, especially attend patient or handle the child, who has been about an infectious case (see list), unless they have disinfected themselves and clothing.

The nurse should refuse to attend an obstetric case if she is suffering with any eruption or purulent discharge from any source, and be extremely careful during her menstrual period, especially if she has any uterine disease.

The nurse should see that the bed is properly arranged and clean, the mother bathed, the external genital organs thoroughly washed with soap and warm water before labor, *after bowels have been moved and bladder emptied.*

Have plenty of hot water, *clean* towels, washbowl, soap, *clean* cloths, scissors, spool of white thread or piece of twine, babe's clothing and a change of clean clothing for mother at hand, so that no time will be lost hunting and preparing them when needed. The *nurse's hands* should be washed and disinfected every time before changing the dressings or cleansing the genital organs of the mother. It is also very important that this disinfection and cleansing of the hands be done after attending mother at stool, changing the baby, and especially after *attending to the cord when suppurating. No vaginal douche should be used during nor after labor except by special instruction of the physician.*

Everything about the bed should be kept scrupulously clean; anything soiled with discharges or blood should be promptly removed.

If antiseptic occlusion pad is used the bandages, etc., should be changed when soiled, and dressings burned as soon as removed.

### MOTHER,

When pregnant, should place herself under the care of a competent physician so he may recognize and treat any complications that may arise. When any of the following occur during pregnancy the physician should be notified: Hemorrhage, obstinate constipation, severe leucorrheal discharge; any special disturbance of kidneys; swelling of the extremities, especially puffiness of face and eyelids; severe headache with disturbance of vision, with or without nausea; any eruptions on body or genitalia; fever with or without chilly sensations; death of the fetus *in utero*.

The mother during pregnancy should take exercise daily—best in the open air by walking. Doing nothing physically and mentally is injurious to both mother and child; while exercise is essential to good health and proper development; yet violent muscular exertion should be avoided. Regularity should be the guiding principle throughout pregnancy. Regular exercise, regular meals, regular sleep, regular bathing, regular daily evacuation of bowels, regulation of kind and quantity of food, regulation of dress, avoiding extreme changes, constriction of waist or pressure on breasts, keeping extremities and feet warmly clad in cold weather, regulation of mental thought, avoiding mental excitement, prolonged mental depression and irritable temper. Pursue pleasant lines of thought, seek congenial, elevating associations, live in a high plane of morality in thought, action, words and deeds. Take a tepid bath at least twice each week in cold weather, each day in warm weather. A vaginal douche of a half gallon of water, near temperature of body, containing a tablespoonful of boric acid should be used two or three times a week, if any leucorrhea or irritating discharge be present; use daily if discharge very free. Bowels should be kept moving each day, using a mild laxative if needed, avoiding drastic cathartics. The use of fruits and vegetables will tend to regulate bowels, also drinking freely of hot water one to two hours before meals.

The diet during pregnancy should be of a nourishing, easily digestible character, consisting mainly of fruits, vegetables, cereals, with a reasonable amount of meats, with plenty of milk and eggs.

The use of "Mothers' Friend" or medicines "to soften pelvic bones," or "prevent ossification of bones in fetus," as advised by some, is nonsense, a relic of superstition, and without scientific or reasonable foundation.

For labor have prepared: Chloroform, Squibb's, 2 ounces; boric acid powder, pure, 4 ounces; mercuric chloride tablets, 1 bottle; absorbent cotton, ½ to 1 pound, in one unbroken package; 1 dozen safety pins; 1 cake of Castile soap, best quality; 2 pieces straight unbleached muslin, 1¼ yards long by ½ yard wide, for binder, or best, an old sheet, *washed, boiled and dried;* a lot of clean cloths, *washed, boiled and dried;* $\frac{1}{2}$ to 1 dozen clean sheets; $\frac{1}{2}$ to 1 dozen clean towels; 1 piece rubber or table oil cloth $1\frac{1}{2}$ by 2 yards; 1 labor pad, else clean sheets or quilt, sterilized by boiling, to place under hips.

*Utensils*—Wash-stand; 2 wash-bowls or basins; 2 water pitchers; toilet or *carbolic soap;* slop jar; vessel for after-birth; 1 pair of scissors; hot and cold water; 1 fountain syringe.

### NIPPLES.

*Before Labor*—If none, should be developed by traction with thumb and finger, or a bottle heated with water emptied and mouth placed on nipple. All pressure of clothing, corset, etc., should be removed from nipples. Astringents in alcohol to harden nipples render skin dry and hard and more liable to fissures, causing severe pain and frequently abscesses. If astringents (tannin or alum) are used they should be combined with glycerine, best olive oil, with boric acid, 10 grains of each to the ounce, applying it to nipple twice each day. Using cocoa butter and kneading nipple is to be preferred. All crusts should be removed from nipple and areola before applications. Exposing them to the air has a beneficial effect.

*After Labor*—Nipples should be cleansed each time baby nurses, best with a solution of boric acid one tablespoonful in a pint of water; kept in a clean bottle convenient for the purpose. Washing out baby's mouth with same solution will lessen danger of infecting nipple or breast, especially if any diseased condition of its mouth or disordered digestion be present. To prevent caking or abscess of breast, nipples should be kept aseptic (surgically clean) and breasts thoroughly emptied. If child fails to empty them the kneading process or breast pump should be used. If cakes occur *very cold or very hot* applications should be used to liquefy contents, then emptied. The drawing of the milk by grown people or puppies is a dangerous procedure and liable to infect both mother and baby. Bandages over breasts to prevent refilling when empty should be superintended by the physician; also the application of ice-bags.

